# Genomics and Transcriptomics of 3ANX (NX-2) and NX (NX-3) Producing Isolates of *Fusarium graminearum*

**DOI:** 10.3390/toxins17060284

**Published:** 2025-06-05

**Authors:** Philip L. Walker, Sean Walkowiak, Srinivas Sura, E. RoTimi Ojo, Maria A. Henriquez

**Affiliations:** 1 Morden Research and Development Centre, Agriculture and Agri-Food Canada, Morden, MB R6M 1Y5, Canada; philip.walker@agr.gc.ca (P.L.W.); srinivas.sura@agr.gc.ca (S.S.); 2 Canadian Grain Commission, Grain Research Laboratory, Winnipeg, MB R3T 2N2, Canada; sean.walkowiak@grainscanada.gc.ca; 3 Soil and Agriculture Weather Surveillance, Manitoba Agriculture, Winnipeg, MB R3T 5S6, Canada; timi.ojo@agr.gc.ca

**Keywords:** *F. graminearum*, genomics, transcriptomics, FHB, pathogenicity, 3ANX, NX

## Abstract

Fusarium head blight (FHB) is an important fungal disease caused by *Fusarium graminearum* and other *Fusarium* spp., resulting in significant yield losses across cereal grains. Recently identified *F. graminearum* isolates in Canada, capable of producing type A trichothecene mycotoxins 3ANX (NX-2, 7-α hydroxy,15-deacetylcalonectrin) and NX (NX-3, 7-α hydroxy, 3,15-dideacetylcalonectrin), demonstrated high levels of genetic diversity. While recent studies have detected this genetic and chemical diversity, little is known of the underlying molecular mechanisms and processes influenced by these distinct chemotypes and regional populations. In the current study, we used an -omics approach coupled with high-resolution mass spectrometry to characterize twenty *F. graminearum* isolates collected from five distinct regions across Manitoba. These data identified regional *F. graminearum* populations within Manitoba that demonstrate distinct genomic variation and patterns of gene expression, particularly within pathogenicity-associated processes. Further, we identified genetic variation and differential expression between isolates showing high and low levels of pathogenicity, allowing for the identification of previously characterized and novel putative pathogenicity factors. Lastly, we detected the production of 3ANX and/or NX mycotoxins within the majority of our twenty characterized *F. graminearum* isolates, suggesting the 3ANX chemotype may be more prevalent than previously expected in Canada. Ultimately, these findings highlight the diversity of *F. graminearum* across Manitoba and, more importantly, uncover specific genomic regions and candidate pathogenicity factors influenced by this diversity.

## 1. Introduction

Fusarium head blight (FHB) is a common fungal disease of cereal food crops, including wheat, corn, barley, oats, and other cereals across the globe. In epidemic years, such as 2016, economic losses from FHB have reached an estimated CAD 1 billion in Canada alone [1]. Rising temperatures and humidity levels are expected to result in an increase in FHB disease pressure, potentially leading to more frequent and destructive FHB outbreaks [2,3]. In addition to causing yield loss, kernel infection, and grain contamination with trichothecene mycotoxins, such as deoxynivalenol (DON), 3-acetyl deoxynivalenol (3ADON), 15-acetyl deoxynivalenol (15ADON), and other toxins, it reduces grain quality and is dangerous for consumption [4].

Trichothecene mycotoxins produced by *Fusarium* species can be classified as type A or type B trichothecenes, differentiated by the absence (type A) or presence (type B) of a keto group at C-8 in their chemical structure [5]. Type B trichothecenes have been of primary concern in *F. graminearum* pathogenicity and three major chemotypes were defined based on the type B trichothecene produced: the nivalenol (NIV) producing chemotype and the DON producing chemotypes, which were categorized into two distinct groups based on the acetylated derivatives of DON produced, 3ADON or 15ADON [6]. Prior research has suggested *Fusarium* strains were limited to producing either DON or NIV [7]; however, recent findings have identified that NIV-producing strains were still capable of producing trace amounts of DON [8].

Historically, 15ADON *Fusarium* isolates have dominated in North America, while 3ADON isolates were the primary chemotype found in both Europe and Asia [9]. However, around the year 2000, the 3ADON chemotype emerged in North America, specifically in the Maritimes, western Canada, and the northern Midwest of the USA [10,11]. For example, in western Canada (Manitoba, Saskatchewan, and Alberta), the 3ADON chemotype currently represents more than 70% of all *F. graminearum* isolates [11,12,13]. The isolates that produce 3ADON generally demonstrate increased production of trichothecene mycotoxins, as well as increased growth rates and spore production, which has been hypothesized to improve fitness and be responsible for the rapid emergence of the 3ADON chemotype in these regions [11]. Direct competition between isolates from the 3ADON and 15ADON chemotypes indicates that some isolates that produce 3ADON are more aggressive/competitive, which could be linked to genome and transcriptome differences between the isolates [14]. Interestingly, regions where the initial emergence of the 3ADON chemotype was identified were also the locations where the novel type A trichothecene chemotype, 3-acetyl NX toxin (3ANX), was discovered in 2010 and has subsequently been predominantly found [11,15,16]. The *F. graminearum* isolates that produced 3ANX were initially thought to be unable to produce NIV and DON, or their acetylated derivatives (3ADON or 15ADON), and were thought to produce only the type A trichothecene 3ANX [15]. However, further characterization of Canadian isolates producing 3ANX has shown 3ANX production in the *F. graminearum* isolates that also produce 15ADON [9], as well as 3ANX and NX production in both 3ADON and 15ADON isolates [17]. The production of 3ANX in *F. graminearum* has been associated with specific amino acid residues in the *TRI1* sequence that distinguish 3ANX producers from non-producers [18]; however, 3ANX production by the closely related *Fusarium culmorum* has been shown to occur independent of the presence of this *TRI1* variant, as well as simultaneously with the production of DON and NIV [8]. The production of 3ANX and its deacetylated form, NX, both demonstrate cytotoxicity through protein synthesis inhibition. A recent intestinal toxicity study [19] found the deacetylated NX to have the greatest impact on intestinal health, followed by DON, then 3ANX. Due to the recent emergence of 3ANX and NX producing chemotypes and their high levels of toxicity, which have been reported to be higher than DON, there is a need to improve our understanding of the prevalence and pathogenicity of this 3ANX chemotype [20,21].

*F. graminearum* population studies performed across the Canadian prairies have estimated high levels of gene flow and low genetic diversity within Manitoba isolates, as well as between isolates across Manitoba and Saskatchewan [13,22,23]. Understanding this genetic diversity and/or consistency between populations is key to developing proper management strategies and predicting potential future outbreaks. Genetic diversity can be indicative of rapid evolution in pathogenic fungi that are able to overcome resistant cultivars, fungicide applications, and other management strategies [13]. The growing conditions in Manitoba often experience high moisture, which is highly conducive to FHB development, and, as a result, the incidence of FHB is typically higher in Manitoba than in other regions [24]. Manitoba is also prone to flooding, which could result in the spread and introduction of diverse plant pathogens from other regions. Due to the high risk of FHB in Manitoba, fungicide applications and the use of FHB-moderately resistant (MR) varieties like AAC Brandon are management strategies that may also increase the risk of developing new genetically diverse and highly pathogenic strains of *F. graminearum* as a response to these FHB management strategies [13,25]. Prior genomic analyses of *F. graminearum* isolates have allowed for the identification of genomic locations rich in diversity, which were helpful in identifying the candidate genes associated with evolutionary fitness [26,27]. The identification of unique and conserved gene variants across distinct *F. graminearum* isolates that differ in their pathogenicity could ultimately improve our understanding of *F. graminearum* pathogenicity. Transcriptomics has also provided insight into *F. graminearum* pathogenicity, using in vitro and in vivo grown *F. graminearum* to identify putative pathogenicity factors induced throughout FHB infection of both FHB-susceptible and FHB-resistant cultivars [14,28,29]. Together, these -omics approaches can predict underlying molecular mechanisms associated with pathogenicity and reveal key distinctions between pathogenic and non-pathogenic *F. graminearum* isolates.

In this research, we profiled the genome and transcriptome of twenty *F. graminearum* isolates across Manitoba. Here, we identified significant differential expression and unique genomic signatures associated with regions in Manitoba, as well as between 3ADON and 15ADON chemotypes. Further, we identified 3ANX and/or NX production in over half of our *F. graminearum* isolates characterized concurrently with DON, 3ADON, 15ADON, and in some cases NIV, as well.

## 2. Results

### 2.1. F. graminearum Isolate Growth and Pathogenicity

**Figure 1:**
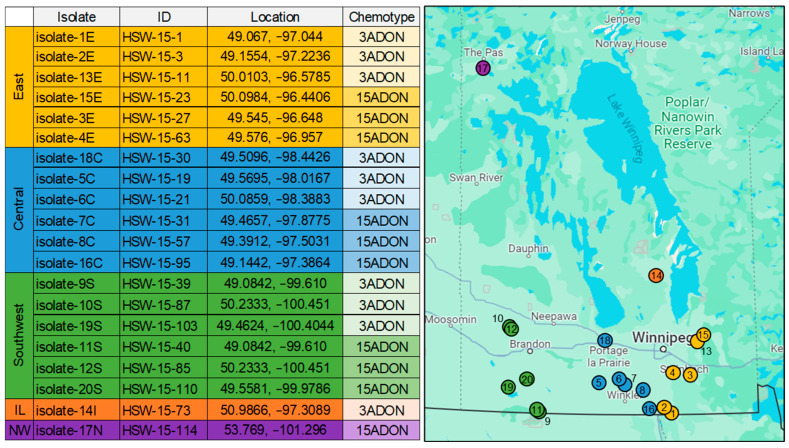
*Fusarium graminearum* isolate chemotypes and collection locations. Isolates were collected from wheat spikes collected between late July and early August of 2015 at growth stage ZGS 73–85. Isolate species and chemotypes were confirmed using PCR.

**Figure 2:**
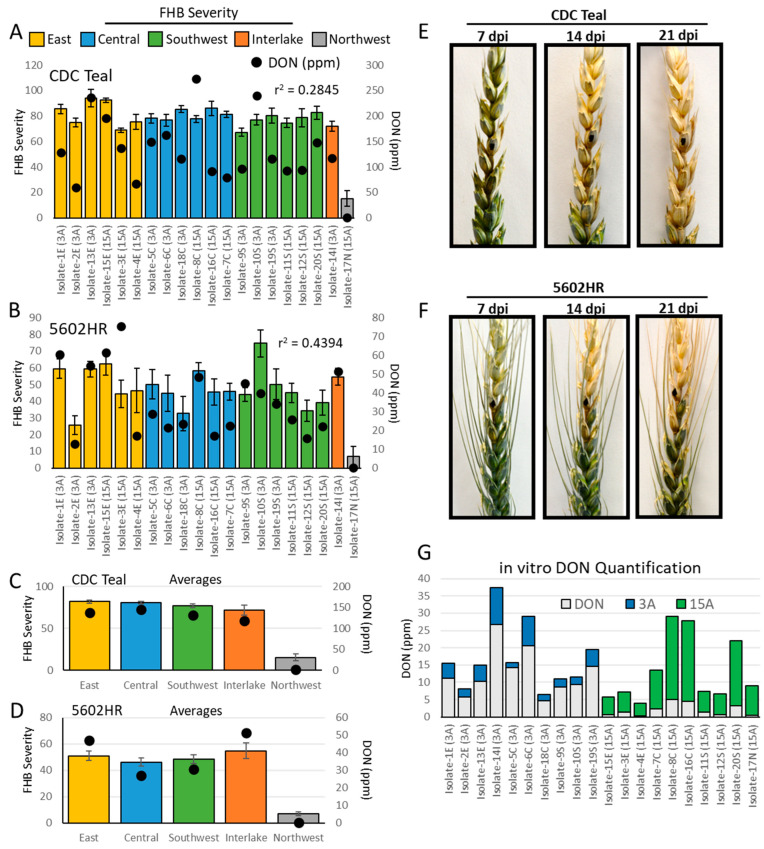
Pathogenicity and DON quantification of the *Fusarium graminearum* isolates. (**A**) The FHB severity quantified in CDC Teal and (**B**) 5602HR at 21 dpi using the point inoculation method (50,000 spores/mL) coupled with DON quantification, using high-resolution mass spectrometry (HRMS). (**C**) The average FHB severity and DON content in CDC Teal and (**D**) 5602HR. The disease progression at 7, 14, and 21 dpi in (**E**) CDC Teal and (**F**) 5602HR. (**G**) The DON content quantified in vitro in rice media, using HRMS. Black dots represent DON content.

All twenty *F. graminearum* isolates (Figure 1) demonstrated high levels of pathogenicity infecting FHB-susceptible CDC Teal at 21 days post inoculation (dpi), with the exception of the non-pathogenic isolate-17N (Figure 2A). In contrast, multiple isolates demonstrate low levels of pathogenicity when infecting the moderately resistant variety 5602HR (Figure 2B). Here, isolates -2E, -12S, -17N, and -18C displayed an FHB severity under 35%, while the remaining isolates showed an average severity of 52 ± 7.5 (SE) (Figure 2B). Quantification of DON revealed little correlation between the FHB severity and DON content within the isolates in CDC Teal (r^2^ = 0.28); however, we observed a moderate positive relationship (r^2^ = 0.44) in 5602HR pathogenicity tests (Figure 2A,B). Further, the non-pathogenic isolate-17N showed essentially no infection across both CDC Teal and 5602HR, coupled with no DON production (Figure 2A,B). The distinct geographical locations (east, central, and southwest) of isolates in Manitoba showed only marginal differences in pathogenicity in both CDC Teal and 5602HR varieties, with average FHB severity values of 81.9 ± 2.1, 80.9 ± 1.6, and 76.7 ± 2.1, respectively, in CDC Teal, and 50.9 ± 3.6, 46.2 ± 3.0, and 48.3 ± 3.4, respectively, in 5602HR (Figure 2C,D). One isolate (isolate-14I) also demonstrated similar FHB severities (72.0 ± 6.0 in CDC Teal and 54.7 ± 6.1 in 5602HR); however, these data represent bio-replicates from a single isolate within this region (Figure 2C,D). Similarly, only one isolate was collected from the northwest region, the non-pathogenic isolate-17N, showing a FHB severity of 15.1 ± 4.1 and 7.0 ± 1.2 in CDC Teal and 5602HR, respectively (Figure 2C,D). The DON, 3ADON, and 15ADON content was also quantified in vitro using rice media for each isolate, where DON concentrations were significantly higher (average = 12.6 ppm ± 3.2) in 3ADON-producing isolates compared to 15ADON producers, which showed little (average = 2.0 ppm ± 0.5) DON production (Figure 2E).

### 2.2. Variant Identification and Differential Expression of Pathogenicity Factors in F. graminearum Isolates

**Figure 3:**
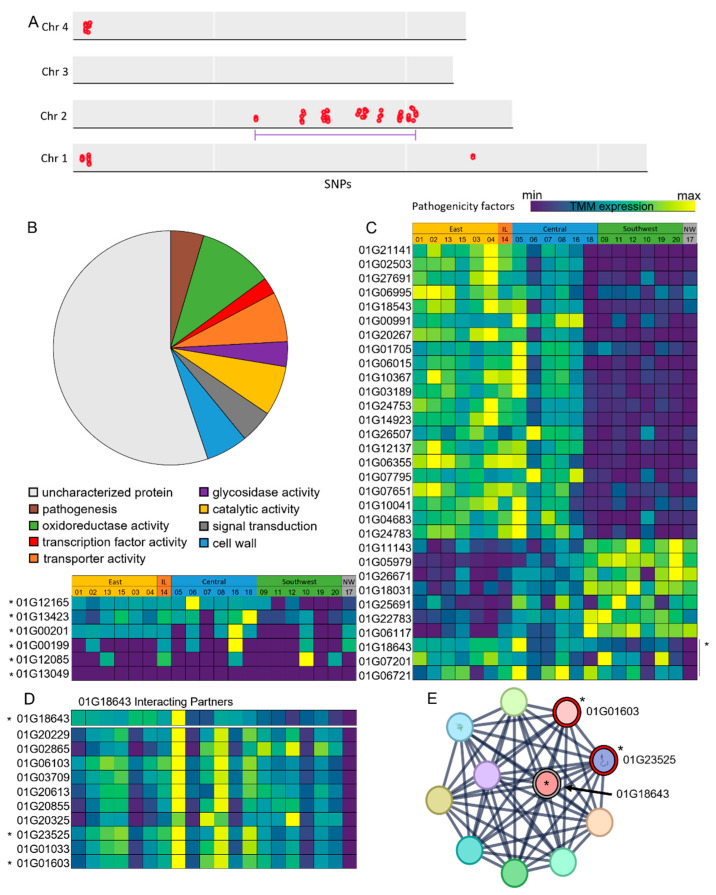
Variant identification and differential expression of putative pathogenicity factors. (**A**) Conserved variants detected within isolates showing reduced pathogenicity using BCFtools and (**B**) enriched GO terms within genes containing modifier variants in section A. (**C**) Trimmed mean of M-values (TMM) expression heatmap of putative pathogenicity factors plotted across isolates using EdgeR. (**D**) Expression of FGRAMPH1_01G18643 predictive interacting partners and (**E**) predictive partner interacting network generated using stringdb. * = gene with modifier variants present. Red dots represent genes with modifier variants at specific loci. Purple underline represents chromosomal region with significant number of variants present.

To better understand differences between highly pathogenic isolates and isolates demonstrating reduced pathogenicity (isolates-2E, -12S, -17N, -18C), we identified differentially expressed genes specific to these isolates, as well as conserved DNA variation belonging to these groups (Figure 3A–D). The conserved variants were predominantly found on chromosome 2, where we identified a statistically significant variant cluster from 2.8 Mb to 6.4 Mb (Figure 3A). Further, two clusters of variants were identified at the 0.15–0.38 Mb regions of chromosomes 1 and 4 (Figure 3A). GO analysis of the genes modified by these variants revealed associations with oxidoreductase activity (10%), catalytic activity (7%), transporter activity (7%), cell wall (6%), pathogenesis (5%), signal transduction (5%), glycosidase activity (3%), and transcription factor activity (2%), while 55% of genes identified encoded uncharacterized proteins (Figure 3B). Within this gene set, we also uncovered previously identified pathogenicity factors, including two small-secreted cysteine-rich proteins (SSCPs) (*FGRAMPH1_01G00199* and *FGRAMPH1_01G00201*), a putative PR-1 effector (*FGRAMPH1_01G12085*), a laccase involved in lignin biosynthesis (*FGRAMPH1_01G13049*), and *FGRAMPH1_01G13423*, which is essential to salicylic acid degradation during FHB infection [30]. We further plotted the in vitro expression of these pathogenicity factors using our RNA-Seq data and identified reduced expression in the southwest isolates relative to the east and central groups in SSCPs *FGRAMPH1_01G00199* and *FGRAMPH1_01G00201*, and the PR-1 effector *FGRAMPH1_01G12085* (Figure 3B). However, no definitive pattern of expression was identified across our isolates, as previous data suggests the expression of these pathogenicity factors is typically induced throughout infection and only expressed at low levels in vitro [31]. We further plotted the in vitro expression of 31 previously identified and putative pathogenicity factors. Here, we found 24 of these genes followed an expression pattern of increased expression in the east isolates, followed by a dampening in the central isolates, and a significantly reduced expression in our southwest isolates (Figure 3C). However, we also found 7 pathogenicity factors that demonstrate an inverse expression pattern, with high expression levels in southwest-located isolates and reduced expression in the east isolates (Figure 3C). Further, three pathogenicity factors (mRNA binding protein *VR1*-*FGRAMPH1_01G18643*, *FGRAMPH1_01G07201*, and *FGRAMPH1_01G06721*) showed expression across all isolates with the exception of the non-pathogenic northwest isolate-17N (Figure 3C), where *VR1* also contained modifier variants specific to isolate-17N. The presence of modifier variants suggests that the differential expression may be due to sequence variation relative to the PH-1 reference, resulting in fewer successfully mapped reads. Further investigation of the predictive interacting partners of *VR1* showed a similar pattern of expression across these interacting partners, with significantly reduced expression in isolate-17N, while two interacting partners (*FGRAMPH1_01G01603* and *FGRAMPH1_01G23525*) also contained modifier variants specific to isolate-17N (Figure 3D,E).

**Figure 4:**
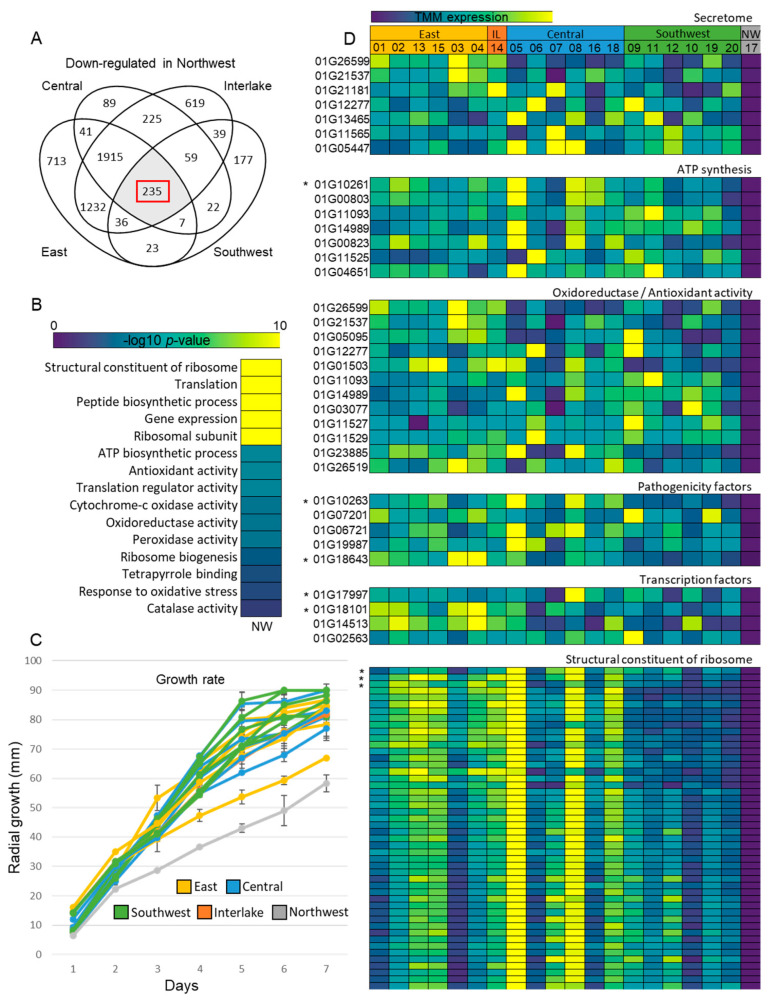
Differential expression analysis between pathogenic *F. graminearum* isolates and non-pathogenic isolate-17N. (**A**) Significantly down-regulated genes in non-pathogenic isolate-17N detected using DESeq2 with an adjusted *p*-value cut-off of *p* < 0.01. (**B**) Gene ontology (GO) enrichment of highlighted shared down-regulated genes in non-pathogenic isolate-17N performed using ShinyGO. (**C**) Growth rate determined by measuring radial growth at 24 h intervals for 7 days in carrot agar media. (**D**) Trimmed mean of M-values (TMM) expression heatmaps of significantly down-regulated genes in isolate-17N. * = gene with modifier variants present.

To further explore the underlying mechanisms of pathogenicity, we identified significantly differentially expressed genes (DEGs) between our non-pathogenic isolate-17N and the remaining pathogenic isolates, where 235 DEGs were significantly down-regulated in isolate-17N (Figure 4A). Of the 235 DEGs, 11 demonstrated modifier variants present within the gene sequence, again suggesting that differential expression within these genes may stem from allelic differences relative to PH-1 (Figure 4D). GO analysis of the 235 DEGs revealed the enrichment of the structural constituent of ribosome (*p* = 2.30 × 10^−73^) and peptide biosynthesis (*p* = 3.61 × 10^−62^), ATP biosynthesis (*p* = 0.0031), oxidoreductase (*p* = 0.0094), peroxidase (*p* = 0.0092), and antioxidant (*p* = 0.0033) activity (Figure 4B), as well as previously identified pathogenicity factors and multiple genes encoding secreted proteins (Figure 4D). Further, we identified several genes containing modifying variants specific to isolate-17N within our down-regulated DEGs, including the pathogenicity factors *VR1* and *FgNACα* (*FGRAMPH1_01G10263*), transcription factors *Zn2Cys6* (*FGRAMPH1_01G17997*) and *MYB10* (*FGRAMPH1_01G18101*), ribosomal genes 40S ribosomal S6-B (*FGRAMPH1_01G01603*), 37S mitochondrial ribosomal protein (*FGRAMPH1_01G04031*) and 60S ribosomal protein L14-B (*FGRAMPH1_01G10265*), and mitochondrial ATP synthase subunit d (*FGRAMPH1_01G10261*) involved in ATP synthesis (Figure 4D). As we discovered significant down-regulation of protein synthesis and other essential biological processes, we plotted the in vitro growth rate of our twenty isolates, monitoring growth every 24 h for 7 days (Figure 4C). Here, we found the non-pathogenic isolate-17N displayed the slowest growth rate of all twenty isolates, showing reduced growth at every 24 h period recorded (Figure 4C). These data also showed no significant differences in isolate growth rate between geographical locations (Figure 4C).

### 2.3. Regional F. graminearum Populations Show Distinct Expression Patterns and Conserved Genomic Variants

**Figure 5:**
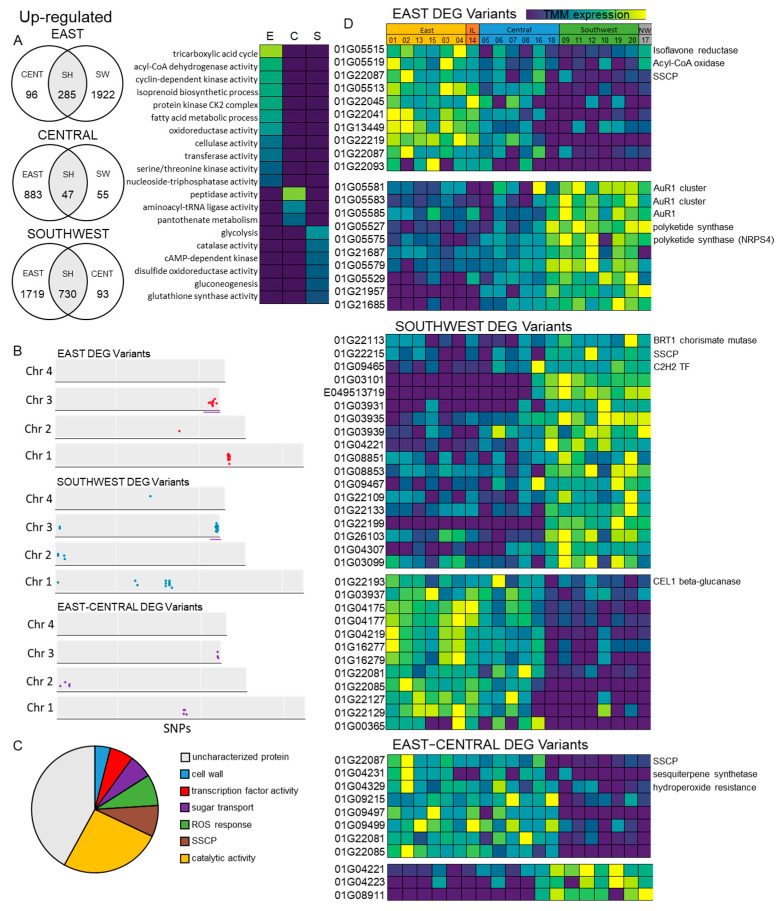
Differential expression and variant identification between isolate collection regions. (**A**) Significantly up-regulated genes detected between east, central, and southwest isolate regions using DESeq2 (*p* < 0.01), and GO enrichment on up-regulated genes using ShinyGO. (**B**) Variants identified specific to east and southwest regions, as well as conserved between east and central regions, using BCFtools. Colored dots represent genes with modifier variants at specific loci. Purple underline represents chromosomal region with significant number of variants present. (**C**) Enriched GO terms within genes containing modifier variants. (**D**) Trimmed mean of M-values (TMM) expression heatmaps of DEGs that also contain variants between regions.

We also explored conserved variants and differential expression between regions within Manitoba, comparing the east, central, and southwest collected isolates. Principal component analysis (PCA) of these expression data revealed clustering of isolates primarily based on region, with the southwest and northwest isolates forming a cluster distinct from the east, central, and interlake cluster (Figure S1). We further identified distinct 3ADON and 15ADON clusters within the east, central, and interlake samples, suggesting differential expression between 3ADON and 15ADON chemotypes within these regions (Figure S1). Differential expression analysis of the east, central, and southwest isolates further revealed these regional expression patterns, with the majority of DEGs identified between the east and southwest isolates, supporting the hypothesis that these distal regions were the most distinct from one another (Figure 5A). GO analysis of differentially expressed genes between the east, central, and southwest isolates showed enrichment of the TCA cycle (*p* = 1.37 × 10^−4^), isoprenoid biosynthesis (*p* = 2.50 × 10^−3^), oxidoreductase (*p* = 5.80 × 10^−3^), and cellulase activity (*p* = 3.30 × 10^−2^) specific to the east isolates, peptidase (*p* = 2.43 × 10^−4^) and aminoacyl-tRNA ligase activity (*p* = 1.70 × 10^−2^) in the central isolates, and glycolysis (*p* = 9.51 × 10^−3^), gluconeogenesis (*p* = 5.10 × 10^−2^), and catalase activity (*p* = 4.50 × 10^−2^) specific to the southwest isolates (Figure 5A). Further, we explored the genomic variants identified within the east, central, and southwest regions, where we found a significant cluster of variants at the distal region of chromosome 3 from 7.0 Mb to 7.5 Mb in both the east and southwest isolates (Figure 5B). To complement these data, we performed a GO analysis of the genes containing these modifier variants and identified the enrichment of cell wall associated genes (4%), transcription factors (6%), ROS response (8%), SSCPs (8%), catalytic activity (26%), and sugar transport (6%). However, similarly to the GO analysis of isolate-17N-specific DEGs, a significant number of these variants were within uncharacterized proteins (42%) (Figure 5C).

Next, we plotted the expression of these identified variants, which also displayed significant differential expression between regions. Here, we identified up-regulated genes in the east region, including SSCP *FGRAMPH1_01G22087*, isoflavone reductase *FGRAMPH1_01G05515,* and acyl-CoA oxidase *FGRAMPH1_01G05519*, while down-regulated genes included *AUR1* cluster genes (*FGRAMPH1_01G05585*, *FGRAMPH1_01G05583*, and *FGRAMPH1_01G05581*) and polyketide synthases (*FGRAMPH1_01G05527* and *NRPS4*–*FGRAMPH1_01G05575*) (Figure 5D). Further, the southwest DEG variants included the up-regulated genes *BRT1* chorismate mutase (*FGRAMPH1_01G22113*), SSCP (*FGRAMPH1_01G22115*), C2H2 transcription factor *FGRAMPH1_01G09465*, and the down-regulated *CEL1* β-glucanase (*FGRAMPH1_01G22193*) (Figure 5D). As the east and central collection regions were in close proximity and demonstrated clustering based on expression patterns, we also plotted variants within DEGs identified specifically in the east and central isolates relative to the southwest. Here, we identified up-regulated genes, including the SSCP *FGRAMPH1_01G22087*, the sesquiterpene synthetase *FGRAMPH1_01G04231,* and a hydroperoxide resistance gene *FGRAMPH1_01G04329* (Figure 5D).

### 2.4. Variant Identification Between 3ADON and 15ADON Chemotypes

**Figure 6:**
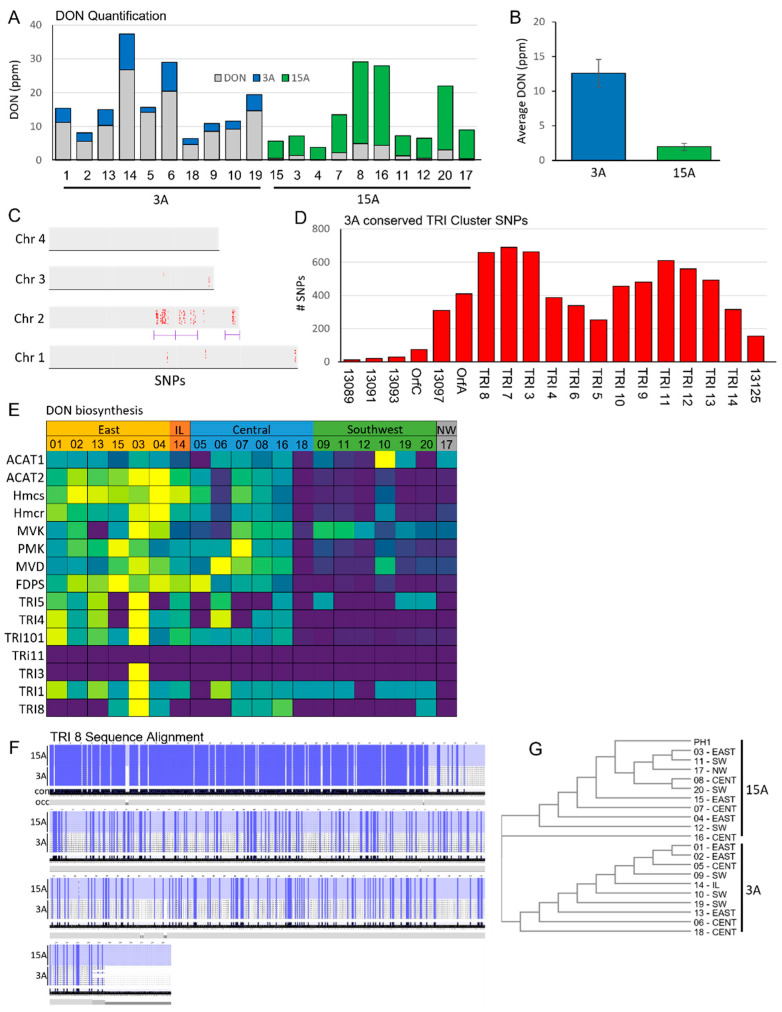
DON quantification and variant identification between the 3ADON and 15ADON chemotypes. (**A**) DON, 3ADON, and 15ADON quantification of *F. graminearum* isolates grown in vitro in rice media using HRMS. (**B**) The average DON content between the 3ADON and 15ADON isolates. (**C**) The conserved variants identified within all 3ADON isolates relative to 15ADON. Red dots represent genes with modifier variants at specific loci. Purple underline represents chromosomal region with significant number of variants present. (**D**) The 3ADON variants identified within the TRI cluster. (**E**) TMM expression heatmap of the DON biosynthesis pathway across the isolates. (**F**) Multiple sequence alignment and (**G**) cluster tree of the TRI8 gene sequence across isolates.

The DON content, as well as 3ADON and 15ADON content, was plotted across our twenty *F. graminearum* isolates to confirm their chemotypes (Figure 6A,B). Here, we confirmed that our ten 3ADON and 15ADON chemotypes were producing 3ADON and 15ADON, respectively. The DON content was significantly higher in our 3ADON isolates, with an average DON ppm of 12.59 ± 2.04 in 3ADON and 1.96 ± 0.51 in 15ADON (Figure 6B). Variant analysis across 3ADON relative to 15ADON chemotypes identified a large number of chemotype-specific variants, with the large majority (98.7%) of these variants being located on chromosome 2, with a significant cluster forming from 5.09 Mb to 6.95 Mb. This variant cluster showed overlap with variants conserved within our reduced pathogenicity isolates and also contained the TRI cluster, where we identified a total of 6915 variants (Figure 6C,D). However, the expression of TRI cluster genes did not show differential expression between 3ADON and 15ADON, but rather showed differential expression based on region (Figure 6E), with the exception of *TRI8*, which only showed expression in 15ADON-producing isolates, which plays a key role in 3ADON/15ADON determination [9,32]. This differential expression is likely due to allelic differences between 3ADON and 15ADON producers, resulting in a lack of *TRI8* mapping in 3ADON producers, which is supported by the PH-1 *F. graminearum* reference isolate being a 15ADON producer [33]. Further, we performed a multiple sequence alignment of the *TRI8* sequence, showing distinct sequence conservation between the 3ADON and 15ADON isolates (Figure 6F,G).

### 2.5. Mycotoxin Profiling Using High-Resolution Mass Spectrometry

**Figure 7:**
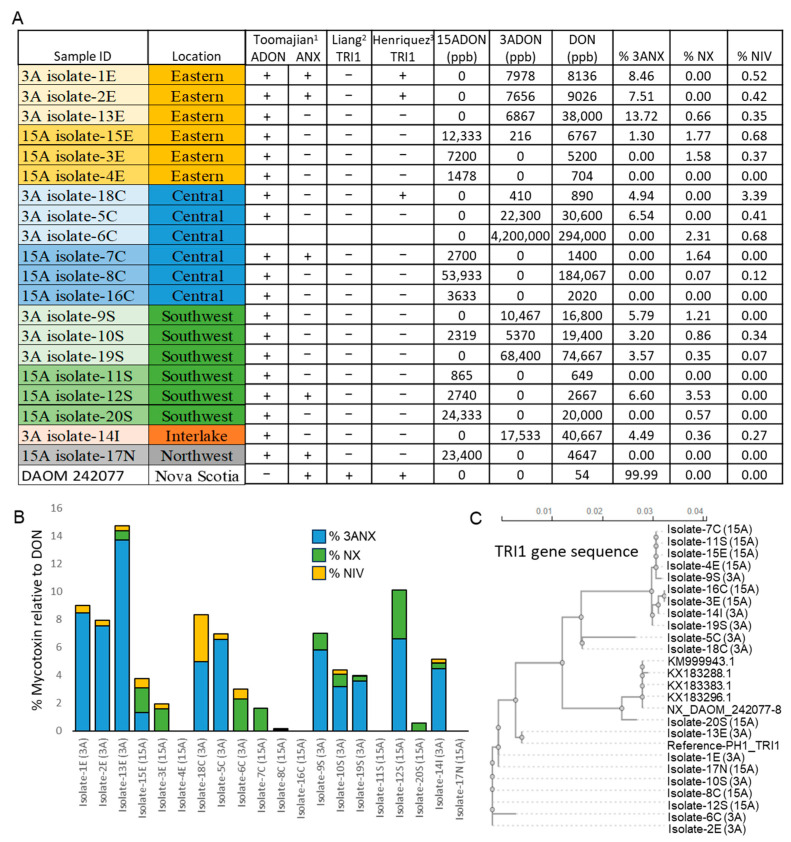
The mycotoxin profile of the *F. graminearum* isolates. (**A**) Mycotoxin (DON, 3ADON, 15ADON, 3ANX, NX, and NIV) analysis performed using HRMS across all *F. graminearum* isolates. (**B**) The percent mycotoxin content relative to DON per isolate. (**C**) A multiple sequence alignment cluster tree of the TRI1 gene sequence per isolate, including the positive NX-2 controls (KM999943.1, KX183288.1, KX183383.1, KX183296.1, and DAOM 242077). ^1^ [9], ^2^ [34], ^3^ [17].

Mycotoxin content data from the HRMS analysis demonstrated that all twenty *F. graminearum* isolates tested were able to produce DON, while also confirming isolates assigned to the 3ADON (isolate-1E, -2E, -13E, -18C, -5C, -6C, -9S, -10S, -19S, and -14I) and 15ADON (isolate-15E, -3E, -4E, -7C, -8C, -16C, -11S, -12S, -20S, and -17N) chemotypes produced their respective 3ADON and 15ADON mycotoxins. However, both acetylated derivatives of DON were detected in isolates -15E and -10S, which were 15ADON and 3ADON chemotypes, respectively (Figure 7A). Further, NIV was detected in trace amounts relative to DON in several of our isolates across both chemotypes, with the majority in the east and central isolates (Figure 7A). With the recent emergence of 3ANX isolates detected in this region, we also performed previously described 3ANX *TRI1* PCR detection methods, as well as mass spectrometric analyses to explore the accuracy of primer detection methods. The Toomajian assay [9], designed to differentiate ADON and 3ANX chemotypes, detected five of our twenty *F. graminearum* isolates (isolates -1E, -2E, -7C, -12S, and -17N) to be 3ANX producers, while [34] *TRI1* primers detected no 3ANX producing isolates, and lastly [17], primers targeting *TRI1* detected three 3ANX producing isolates (isolates -1E, -2E, and -18C) (Figure 7A). In contrast, the HRMS data revealed that eleven of our twenty isolates produced 3ANX, albeit at significantly lower levels relative to DON. The 3ANX positive control isolate DAOMC242077 showed the opposite trend, producing significantly more 3ANX than DON (Figure 7A). Further, we also quantified NX production in our isolates, where seven of the eleven 3ANX-producing isolates also produced trace amounts of NX (Figure 7A). Interestingly, we also identified four isolates that produced NX without 3ANX being detected (Figure 7A). Lastly, we performed a multiple sequence alignment of *TRI1* gene sequences from our *F. graminearum* isolates along with previously identified NX-positive isolates containing the previously described *TRI1* NX-2 allele [18,35]. While our isolates did form two distinct clusters when performing multiple sequence alignment of *TRI1*, neither cluster showed similarity to the 3ANX positive controls, and these clusters did not correlate with NX positive or negative HRMS data (Figure 7C).

## 3. Discussion

During the emergence of 3ADON isolates in North America, multiple reports have described an increase in DON production and disease severity in kernels infected with isolates from the 3ADON chemotype relative to the 15ADON chemotype [36,37]. However, more recent studies have primarily reported increased DON production coupled with no significant differences in FHB severity in 3ADON isolates [38,39]. Population studies specific to Manitoba have also shown increased DON content by 3ADON-producing isolates, with no significant differences in aggressiveness among 3ADON and 15ADON isolates, challenging both FHB-susceptible and FHB-MR wheat varieties [10]. Our Manitoba in vivo collected data also revealed no significant differences in the FHB severity among 3ADON and 15ADON isolates in both FHB-susceptible and FHB-MR wheat; however, the DON content in infected kernels also showed no significant differences between these chemotypes (Figure 2A,C). This agrees with other studies that reported similar levels of mycotoxins in harvest samples containing either or both chemotypes, albeit in years of low infection; however, this may be in part due to the signal from individual isolates being lost in the noise from larger bulked grain samples, when compared to targeted studies performing point inoculations of single isolates [12,24]. The differences in quantification may also be due to the difficulty in quantifying DON production in vivo, with potential masking of DON occurring through host glycosylation in response to infection [40]. DON quantification has proven particularly problematic in wheat varieties with some level of FHB-resistance, like 5602HR, as they are typically more efficient at DON glycosylation in response to infection [41]. In contrast to the in vivo data, in vitro-grown 3ADON isolates did show an increase in DON production relative to 15ADON isolates (Figure 2E). This in vitro quantification may be more representative of these isolates’ ability to produce DON, as host glycosylation cannot influence quantification. Further, increased DON production during infection of the FHB-susceptible CDC Teal compared to FHB-moderately resistant 5602HR was consistent with prior findings, where increased DON content has been found in more susceptible cultivars [42]. However, variability in this correlation has been shown to be influenced by the method of inoculation [43].

There was no significant correlation between the DON content and the pathogenicity across all isolates tested; however, conserved variants in the isolates with reduced or no pathogenicity were identified (Figure 3A). These variants were predominantly located on chromosome 2, with a statistically significant variant cluster ranging from 2.8 Mb to 6.4 Mb (Figure 3A). This region has not only been previously characterized as showing high levels of genetic diversity, but also contains an overrepresentation of genes belonging to the pan-secretome, encoding proteases, CAZymes, and effectors that perform key functions throughout *F. graminearum* pathogenicity [26]. Further, biosynthetic gene clusters involved in virulence, such as the TRI cluster, fall within this region, with 12 of the 15 TRI genes being located within this region on chromosome 2 [26,44]. These secreted protein clusters (SPCs) have previously been described to show high levels of genetic diversity, allowing pathogens to evolve against emerging host defences [26,29]. Within this variant cluster on chromosome 2, we identified genes that encode four secreted proteins involved in host cell wall degradation, including *FGRAMPH1_01G13999* and *FGRAMPH1_01G14001* acting on hemicellulose, the predicted laccase *FGRAMPH1_01G13049* acting on lignin, and *FGRAMPH1_01G12165* acting on cellulose and showing significant down-regulation in hypovirulent *F. graminearum* [29,45]. Further, we were able to identify two SSCPs, *TOX2* and *TOX3* (*FGRAMPH1_01G00199* and *FGRAMPH1_01G00201*), which are necrotrophic effectors highly induced during *F. graminearum* infection that initiate host programmed cell death during pathogenesis [46]. These genes, as well as the several uncharacterized genes identified within this variant cluster region, can serve as candidate genes for further characterization as potential pathogenicity factors in *F. graminearum*, as these data suggest this variant cluster may be an important region for evolving pathogenicity factors and adapting to emerging host defences.

To further explore pathogenicity across all isolates, we plotted the expression of previously identified and putative pathogenicity factors in *F. graminearum* (Figure 3C). As many of these genes have been characterized to be highly induced during infection, our in vitro expression data were unable to identify any expression patterns correlating with isolate pathogenicity. However, we did uncover three pathogenicity factors (*VR1*-*FGRAMPH1_01G18643*, *FGRAMPH1_01G07201,* and *FGRAMPH1_01G06721*) that showed consistent in vitro expression across all pathogenic isolates, with the exception of the non-pathogenic isolate-17N. Further, *VR1* also contained modifier variants specific to isolate-17N (Figure 3C,D), suggesting reduced expression may be the result of allelic differences in gene sequence relative to our reference PH-1 *F. graminearum,* resulting in a reduced number of mapped reads. *VR1* is an orthologue of Nam8 in *Saccharomyces cerevisiae,* which is an RNA-binding protein involved in the regulation of the oxidative stress response, as well as being linked with hypovirulence and *F. graminearum* growth when infected with mycovirus FgV-ch9 [47,48]. The quantification of the identified predictive interacting partners of *VR1* showed similar expression patterns, with significantly reduced expression specific to isolate-17N, suggesting a potential regulatory role of *VR1* on these predictive interacting partners. While many of these potential interacting partners are uncharacterized, the characterized proteins identified showed association with translation and mRNA processing, specifically belonging to the snRNP family, playing roles in the regulation of alternative splicing.

The significantly down-regulated genes specific to the non-pathogenic isolate-17N also showed a strong enrichment of GO terms associated with ribosome structure, translation, and ATP synthesis (Figure 4A,B). Deficiencies in these important growth and development processes may be responsible for the reduced growth rate in isolate-17N relative to the other isolates, with several studies demonstrating deficiencies in protein synthesis, leading to impaired fungal growth [49,50]. Ribosomal proteins and protein synthesis are also targets of different virulence factors, including DON and other trichothecene mycotoxins [51,52]. Not only did we find strong enrichment of processes associated with protein synthesis in our isolate-17N down-regulated gene set, but we also identified ribosomal genes in this group containing modifier variants specific to isolate-17N (*FGRAMPH1_01G01603*, *FGRAMPH1_01G04031*, and *FGRAMPH1_01G10265*) (Figure 4D). Further, the pathogen and host produce a suite of reactive oxygen species during infection, where, in response to this oxidative stress, both the host and pathogen will act to scavenge these ROS through the peroxisome pathway, deploying superoxide dismutase and catalase enzymes to maintain cellular homeostasis [53,54]. Several genes associated with oxidoreductase and antioxidant activity were also down-regulated in isolate-17N, including the catalase *CAT2* (*FGRAMPH1_01G11527*), involved in ROS scavenging, and the super-oxide dismutase (SOD) gene *SOD4* (*FGRAMPH1_01G23885*) [55], where SODs play a role in surviving high ROS concentrations generated by oxidative burst of host cells in response to infection [53]. *SOD4* has also previously been identified as a target candidate in therapeutics targeting the fungal pathogen *Candida auris* and has been hypothesized to be a promising target as an anti-fungal [56]. Further, a variety of SOD knockout mutants have been shown to demonstrate reduced fungal growth and pathogenicity, being described as essential for virulence across multiple fungi [56,57,58]. We also identified the down-regulation of the cytochrome c oxidase polypeptide V *COX5* (*FGRAMPH1_01G03077*), which is necessary for oxidative phosphorylation, acting as a catalyst to the final step in the mitochondrial electron transport chain [59]. This lack of *COX5* expression may also contribute to the reduced growth and pathogenicity of isolate-17N. Lastly, *FgNACα*, a transcription factor essential for vegetative and pathogenic growth in *F. graminearum,* involved in protein biosynthesis and transport, was also found to be down-regulated, as well as containing modifier variants specific to isolate-17N [60]. Together, these isolate-17N-specific significantly down-regulated genes can serve as down-stream candidate genes for further characterization and provide insight into the processes, which may be essential in targeting to build host resistance to FHB.

As our isolates were collected from wheat fields across Manitoba, we also aimed to uncover unique expression patterns and variants across these distinct regions (Figure 5). The east and southwest isolates showed the highest level of differential expression and genetic variation (Figure 5A,B), which was unsurprising due to the proximity of the east and central collection sites relative to the isolated southwest region (Figure 1). A single significant cluster of variants between the east and southwest DEGs was found at the distal sub-telomeric region (7.0 Mb to 7.7 Mb) of chromosome 3 (Figure 5B). Modified genes within this cluster that also demonstrate significant differential expression between regions include pathogenesis-associated genes, and enriched pathways and processes like the ROS response (8%), catalytic activity (26%), SSCPs (8%), and transcription factor activity (6%). These data reveal distinct genomic and transcriptomic signatures conserved between regions in Manitoba, with the majority of these distinct features being associated with pathogenicity (Figure 5). Further, these data support prior hypotheses that key processes involved in pathogenicity are significant regions of genomic and transcriptomic diversity, even between *F. graminearum* populations in close proximity [61]. This diversity is likely due to the consistent selection pressure on these populations, which are subject to not only unique crop species and varieties but also distinct environments and microclimates [62]. Diversity can be influenced by environmental factors, including temperature, precipitation, and humidity, which can dramatically influence FHB disease incidence and severity [13,63]. For example, high precipitation, relative humidity and warm temperatures during and soon after anthesis can greatly favour FHB development in wheat [64]. Therefore, we plotted these environmental parameters from 2011 leading up to our collection date in 2015 to better understand this genetic diversity, especially in pathogenicity-related genes (Supplementary Materials Dataset S5). On average, the air temperature was slightly elevated (~1 °C) in the east and central regions relative to the southwest in all years from 2011 to 2015 during anthesis (mid-June to August) when FHB is most likely to occur [65]. Whereas, when exploring relative humidity and precipitation accumulation, we found the opposite trend, with a small increase in both parameters in the southwest region, excluding our collection year 2015, where we found higher levels of relative humidity and precipitation in the east region (Supplementary Materials Dataset S5). Due to the proximity and similar climate of these regions, it is unlikely that these modest differences in temperature, relative humidity, and precipitation would yield the genetic and transcriptomic differences identified; however, other factors like soil salinity and atmospheric CO_2_ level, as well as the makeup of crops and varieties used in prior years and in adjacent fields, may also contribute to this diversity [66,67]. The exploration of *F. graminearum* diversity across proximal geographic locations provides insight into the genetic makeup of *F. graminearum* populations and suggests a high level of recombination and genetic plasticity in this region, with variation potentially contributing to differences in pathogenicity, which can ultimately be a risk to current and future FHB-resistant crop varieties [61,68].

Lastly, with the recent emergence of NX-producing 3ANX chemotypes identified in Canada, we examined twenty isolates for NX production using three different 3ANX PCR detection methods, as well as HRMS. We identified several isolates capable of producing trace amounts of 3ANX and/or NX concurrently with DON, where NX producers were primarily identified to be part of members of the 3ADON chemotype (Figure 7A). However, we did find instances of isolates assigned to the 15ADON chemotype producing trace amounts of NX (Figure 7A). This is likely due to the emergence of 3ADON-producing isolates in Canada and the northern United States, which is predicted to correlate with the emergence of 3ANX-producing strains [20,69]. Another possibility for the correlation between 3ADON and NX production could be through the specificity of the *Tri8* allele, which is responsible for generating 3ADON or 15ADON in the DON biosynthetic pathway, while also being proposed to be responsible for producing 3ANX (NX-2) in the NX biosynthetic pathway [5,18]. Due to the structural similarities between 3ANX and 3ADON, the 3ADON *Tri8* allele may demonstrate improved compatibility and efficiency with 3ANX production relative to 15ADON producers [8]. However, a prior study by [9] exploring NX-producing isolates in Ontario, Canada, hypothesized an evolution of these 3ANX isolates from a 15ADON background, as the majority of NX producers identified in this region were 15ADON producers. Further, we were unable to identify any correlation between NX production and pathogenicity; however, this analysis is limited due to our NX-producing isolates only producing trace amounts relative to DON and its acetylated derivatives (Figure 7A). Further, mass spectrometric analysis detected NX-3 in isolates where no NX-2 was detected, likely due to deacetylation of NX-2 to NX-3, similar to the deacetylation of 3ADON to DON [69]. Also, we did not test 3ANX or NX production in planta.

Our data suggest that the previously described 3ANX-associated *TRI1* alleles are not necessary for NX-2 production, as we were unable to identify *TRI1* sequence conservation between previously characterized 3ANX isolates and our NX-producing isolates (Figure 7A–C). While we identified sequence diversity within the *TRI1* allele of our twenty isolates in the form of two distinct clusters, these clusters did not correlate with NX production or with the collection region. Prior studies have suggested a specific *TRI1* allele to be necessary for NX production in *F. graminearum*; however, the closely related species *F. culmorum* produces NX independently of a *TRI1* allele [8]. Here, we have demonstrated this *TRI1*-independent NX production in *F. graminearum*, as NX producers and non-producers showed no conserved distinctions in their *TRI1* sequence (Figure 7C).

Lastly, previously described 3ANX detection methods using PCR to target the NX *TRI1* allele proved to be insufficient in accurately detecting isolates capable of NX production. Mass spectrometry analysis for mycotoxins detected NX in several isolates that tested negative for the NX *TRI1* allele across all three *TRI1* allele detection assays. In addition, these assays also showed variation between one another, with the exception of the Toomajian [9,17] assays, both correctly identifying 3ANX positives in isolates -1E and -2E (Figure 7A). These data demonstrated that prior 3ANX PCR detection methods that are based on the NX *TRI1* allele and most often used may be underestimating the number of NX-producing *F. graminearum* isolates occurring in Manitoba, and likely across Canada and the northern United States, where these isolates have primarily been identified [11,16]. However, it is important to acknowledge that the 3ANX and NX production in the isolates from our study is lower than that of isolates reported to have the *TRI1* allele normally associated with NX and 3ANX production. Further studies are required to identify additional pathways for the biosynthesis of NX and 3ANX in the isolates presented here, as well as for developing genomics tools for the surveillance of field-collected samples.

In conclusion, these data revealed distinct genomic and transcriptomic signatures in *F. graminearum* isolates between regions across Manitoba, as well as between pathogenic and non-pathogenic isolates. Further, mycotoxin profiling using HRMS suggested NX producers may be more prevalent than previously thought, with over 50% of isolates tested showing NX production. These findings ultimately improve our understanding of *F. graminearum* diversity in Manitoba and reveal specific genomic regions and putative pathogenicity factors with high levels of diversity, which could be leveraged to help in the development of successful disease management strategies against FHB and emerging novel mycotoxins.

## 4. Materials and Methods

### 4.1. Culturing of F. graminearum Isolates

Twenty *F. graminearum* isolates (Figure 1) were collected from eighteen distinct wheat-producing farms across Manitoba that covered the east, central, southwest, interlake, and northwest regions (Figure 1). Sampling was performed between late July and early August of 2015, when the majority of wheat was at the ZGS 73–85 growth stage [70]. Wheat spikes were collected, and the kernels were subsequently surface-sterilized and incubated on 25% strength potato dextrose agar (PDA) with 0.02% streptomycin sulphate at 21–22 °C for 7 days under fluorescent light. The fungal isolates investigated in this study were deposited into the M.A. Henriquez culture collection at the Morden Research and Development Centre, Agriculture and Agri-Food Canada, Morden, MB, Canada (HSW-2015). These isolates are available for research purposes upon request.

### 4.2. Plant Material and Disease Assessment

Wheat cultivars used in this study included the spring wheat cultivars CDC Teal, susceptible to FHB [71], and 5602HR, moderately resistant to FHB [72]. Growing conditions for the wheat plants were similar to those used in [73]. Inoculation was performed at 50% anthesis for two florets between the palea and lemma at the middle of the spike, using 10 µL of a macroconidia suspension of *F. graminearum* (5 × 10^4^ macroconidia/mL) and water as a control. Inoculated spikes were enclosed in a plastic zip bag for 48 h to stimulate infection. Two individual wheat plants and four replicates for all cultivars per isolate and water control were evaluated. The experiment was repeated twice. Spikelets demonstrating disease symptoms within each spike were recorded at 7, 14, and 21 dpi to assess type II FHB resistance for a total of 336 wheat heads. The FHB severity was calculated by quantifying the diseased spikelets relative to the total spikelets present per wheat head. The harvested wheat heads were processed for mycotoxin analysis using high-resolution mass spectrometry (HRMS). These data can be found in the Supplementary Materials, Dataset S1.

### 4.3. DNA Extraction

The DNA was extracted according to the protocol described by [17]. Briefly, *F. graminearum* mycelia were macerated in a TES extraction buffer containing 0.2 M Tris pH 7.5, 10 mM EDTA pH 8.0, and 0.5 M NaCl. The supernatant was mixed with 500 µL chloroform–isoamyl alcohol (24:1). The DNA was subsequently precipitated in ice-cold isopropanol and washed with 70% ethanol. The resuspended DNA pellet was quantified using a NanoDrop spectrophotometer 2000 (Thermo Scientific, Waltham, MA, USA). The DNA was then cleaned with the DNeasy PowerClean Pro Cleanup Kit (Qiagen, Venlo, The Netherlands), following the manufacturer’s protocol.

### 4.4. F. graminearum Identification Using PCR

*F. graminearum*-specific PCR was performed using Fg16F/Fg16R primers [74], resulting in a 410 bp amplicon, as described by [17]. The PCR reactions were performed using 0.625 U of Thermo Scientific DreamTaq (Thermo Fisher Scientific, Waltham, MA, USA), 1 × Thermo Scientific DreamTaq Buffer (Thermo Fisher Scientific, Waltham, MA, USA), 0.2 mM dNTPs, 1.2 mM MgCl_2_, 0.8 mg/mL BSA, 0.16 µM per primer, and 20 ng genomic DNA. The PCR was performed using the C-1000 Thermal Cycler (Bio-Rad Laboratories, Hercules, CA, USA), using the following thermal cycling protocol: 3 min at 95 °C, 35 cycles at 95 °C for 30 sec, 55 °C for 30 sec, 72 °C for 1 min, followed by a final extension at 72 °C for 5 min. Gel electrophoresis on 1.5% agarose gels was subsequently used to estimate fragment size, using a 1 kb Plus DNA Ladder (Thermo Fisher Scientific, Waltham, MA, USA).

### 4.5. F. graminearum In Vitro Growth Rate

*F. graminearum* spore suspension (105 spores/mL) was prepared from *F. graminearum* grown on Spezieller Nährstoffar Agar (SNA) media with filter paper for 10 days [75]. A total of 10 µL of *F. graminearum* spore suspension was centre-inoculated onto carrot agar media in 90 mm diameter petri dishes and incubated under fluorescent lighting at 22 °C until the mycelia reached the edge of the plate. The growth rate was determined by measuring the radial growth at 24 h intervals from inoculation until reaching the edge of the plate, with three biological replicates per isolate. The growth rate data can be found in the Supplementary Materials, Dataset S2.

### 4.6. F. graminearum Trichothecene Chemotype Identification

Assignment to the 15ADON, 3ADON, and NIV chemotypes was performed by PCR in 12.5 µL reaction volumes with 1× Thermo Scientific DreamTaq Buffer (Thermo Fisher Scientific, Waltham, MA, USA), 2.0 mM MgCl2, 0.2 mM dNTPs, 0.2 μM of each of the primers 12CON (5′ -CATGAGCATGGTGATGTC-3′), 12NF (5′ -TCTCCTCGTTGTATCTG G-30′), 12-15F (5′ -TACAGCGGTCGCAACTTC-3′), and 12-3F (5′ -CTTTGGCAAGCCCGTGCA-3′) [76], 1.6 mg/mL BSA, 0.5 units of Thermo Scientific DreamTaq (Thermo Fisher Scientific, Waltham, MA, USA), and 20 ng of genomic DNA. The PCR conditions were similar to the above Fg16F/Fg16R primers, with expected products of 670 bp for 15ADON, 410 bp for 3ADON, and 840 bp for NIV.

The PCR-RFLP assay by [34] and the Toomajian assay by [9] were evaluated to identify 3ANX isolates. In addition, the TRI1 allele-specific primers NX-Tri1-F (5′-TCGATGTTAATTGTTTTTGTGTA-3′) and NX-Tri1-R (5′-AGCCAGCTGGGTTTCTTG-3′) [17] were also used to test for 3ANX chemotypes, following the protocol described by [17].

### 4.7. F. graminearum Culture in Rice Media

*F. graminearum* isolates were incubated at 22 °C for 10 d under a combination of fluorescent–UV lights [73]. Rice cultures were subsequently prepared, according to [17], by autoclaving 10 g of washed rice with distilled water in 50 mL glass beakers covered in aluminum foil prior to inoculation with 5 × 10^4^ macroconidia/mL and water (control). Three replicates were incubated for 10 days at 22 °C and freeze-dried for 48 h prior to mycotoxin profiling.

### 4.8. Mycotoxin Profiling Using UHPLC-HRMS

Mycotoxin profiling was conducted following the protocol described in [17]. Briefly, sample extracts were spiked with 10 µL of stable isotope ^13^C-labelled internal standards mix, containing 2.5 µg/mL each (^13^C_15_-DON, ^13^C_17_-3-acetyl DON, ^13^C_17_-15-acetyl DON, and ^13^C_15_ nivenol) prior to analysis. Data was acquired using an ultra-high-performance liquid chromatography (UHPLC) (Vanquish, ThermoFisher, Mississauga, ON, Canada) coupled with (HRMS, Tribrid ID-X, ThermoFisher, Mississauga, ON, Canada).

### 4.9. Whole Genome Sequencing and Genome Assembly

DNA libraries were prepared using the shotgun DNA library preparation (NEB Ultra II), and samples were subsequently sequenced at McGill University and the Genome Quebec Innovation Centre (Montreal, QC, Canada), using the Illumina Shotgun HiSeq 2500 with 125-nucleotide paired-end reads. Illumina TruSeq LT adapters and low-quality reads were trimmed using Trimmomatic v0.36 [77]. Genome assemblies for *F. graminearum* isolates were generated using an ABySS paired-end sequence assembler [78]. Genome assembly statistics can be found in Table S1. Single-nucleotide polymorphisms (SNPs) and indels were identified using the dnadiff tool in the NUCmer program aligned with *F. graminearum* PH-1 ASM24013v3 (Figure S2) [79]. Unique and conserved variants were identified across samples using BCFtools v1.18 software [80]. Variant filtering was also performed in BCFtools with a threshold of (<20) quality and (<10) depth. GO enrichment was performed on coding sequences containing nucleotide sequence variants using ShinyGO v0.82 [81].

### 4.10. RNA Sequencing and Data Processing

RNA was extracted from seven-day-old *F. graminearum* mycelia grown on PDA with streptomycin sulphate using Trizol Reagent (Ambion, Waltham, MA, USA). The Agilent 2100 Bioanalyzer and the Agilent RNA 6000 Nano Kit (Agilent, Beijing, China) were used to evaluate the RNA concentration and quality. The mean RNA Integrity Number (RIN) across the samples was 9.5 out of 10. The cDNA libraries were prepared by using the NEB rRNA-depleted stranded (plant) kit. The samples were sequenced at McGill University and the Genome Quebec Innovation Centre (Montreal, Canada), using the Illumina NovaSeq 6000 with 100-nucleotide paired-end reads.

The RNA-sequencing raw and processed reads can be found at the Gene Expression Omnibus (GSE292521). Low-quality reads and adapter sequences were removed using Trimmomatic v0.36 [77] with the following parameters: (LEADING:3 TRAILING:3 SLIDINGWINDOW:4:20 MINLEN:36). The surviving reads were aligned to the *F. graminearum* PH-1 (ASM24013v3) reference assembly using HISAT2 v2.2.1 [82]. Count data was generated using featureCounts v2.0.5 [83] and can be found in the Supplementary Materials, Dataset S3. The count data was used as input in performing principal component analysis clustering (Figure S1) using DESeq2 [84], as well as identifying significantly differentially expressed genes (DEGs) in DESeq2. DEGs were considered significant at an adjusted *p*-value cut-off of *p* < 0.01 and sorted using InteractiVenn [85]. TMM expression values were calculated using EdgeR [86]. GO enrichment of DEGs and their selection were performed using ShinyGO v0.82 [81] and visualized using the conditional formatting function in Excel. Supplementary Materials, Dataset S4, contains the DEG lists identified.

## Data Availability

The datasets presented in this study can be found in online repositories. The names of the repositories and accession numbers can be found within the article and Supplementary Materials.

## Supplementary Materials

The following supporting information can be downloaded at: https://www.mdpi.com/article/10.3390/toxins17060284/s1. Figure S1. Principle component analysis of 20 *Fusarium graminearum* isolates. Count data generated using featureCounts was used as input and PCA plot generated using DESeq2 in r. Table S1. Assembly statistics for *F. graminearum* isolates. Figure S2. Small nucleotide polymorphisms (SNPs), indels and alignment percentage of genome per *F. graminearum* isolate relative to PH-1. SNPs and indels detected using NUCMER software and alignment performed using Mummer. Datasets S1–S5.

## Abbreviations

FHB	Fusarium head blight
DON	deoxynivalenol
3ADON	3-acetyl deoxynivalenol
15ADON	15-acetyl deoxynivalenol
NIV	nivalenol
DPI	days post inoculation
HRMS	high-resolution mass spectrometry
UHPLC	ultra-high-performance liquid chromatography
GO	gene ontology
DEG	differentially expressed gene
