# Supplementary figures and images for: Genomics and Transcriptomics of 3ANX (NX-2) and NX (NX-3) Producing Isolates of Fusarium graminearum
Source: Toxins (Basel). 2025 Jun 5;17(6):284. doi: 10.3390/toxins17060284 (PMC12197723; doi:10.3390/toxins17060284)

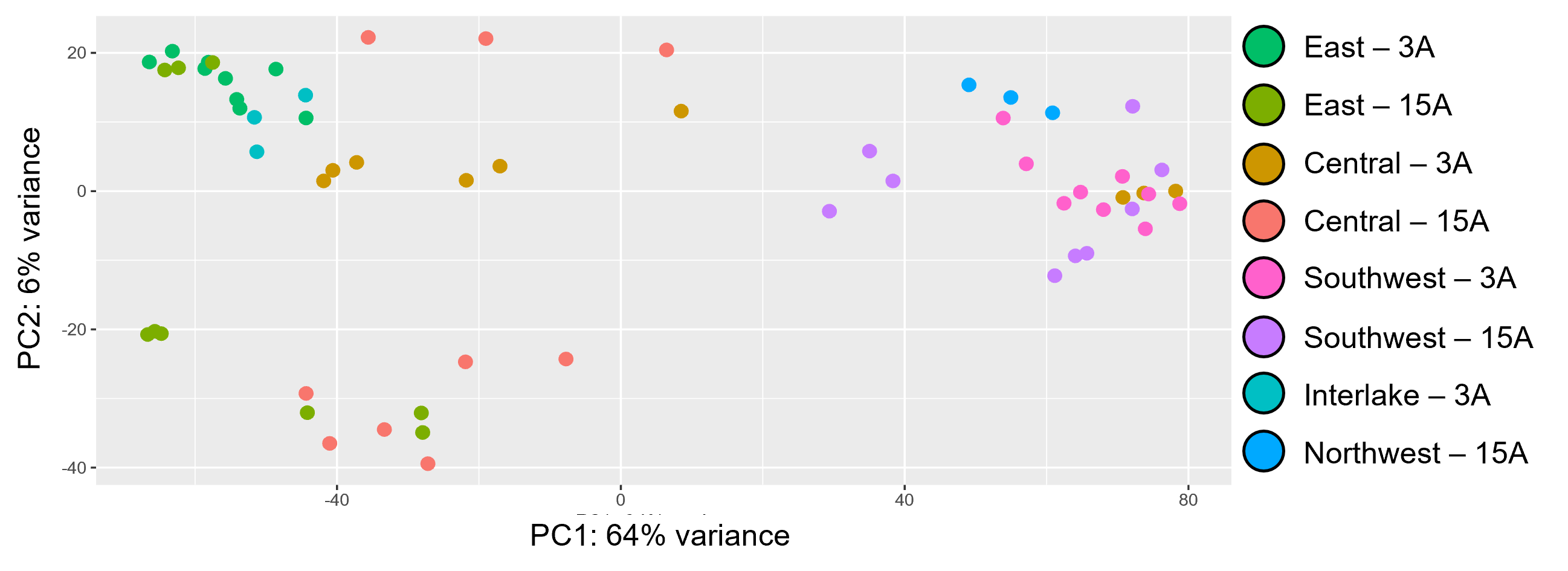

Supplement: Supplementary file 1 [file toxins-17-00284-s001.zip › Figure_S1.tif]

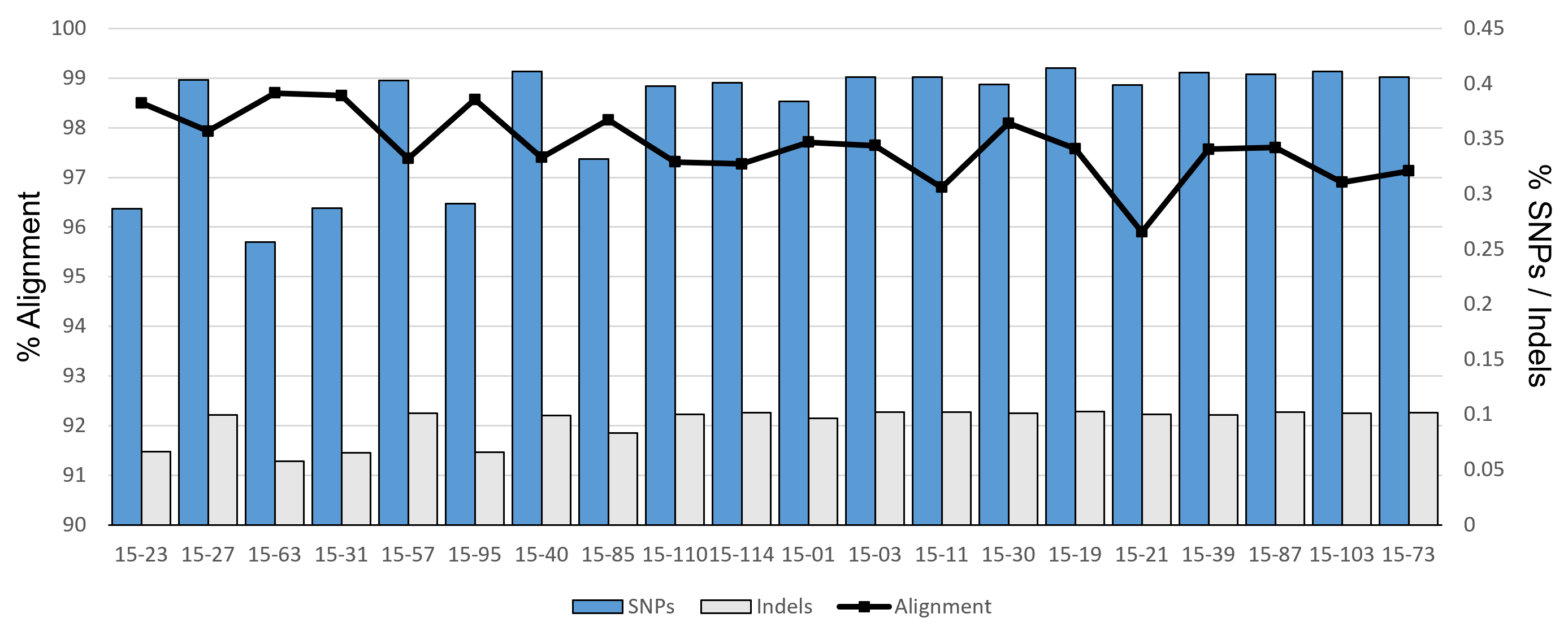

Supplement: Supplementary file 1 [file toxins-17-00284-s001.zip › Figure_S2.tif]

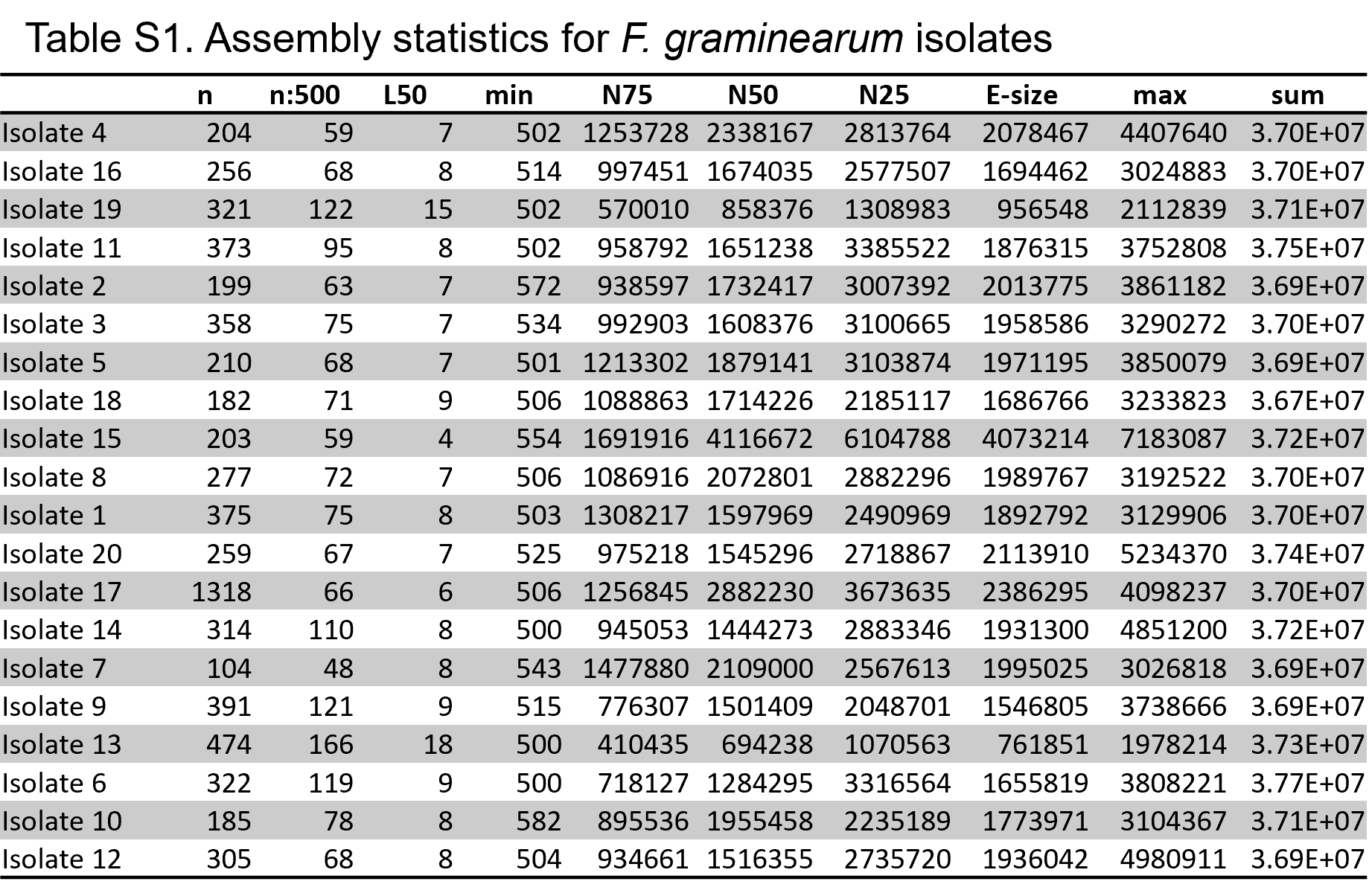

Supplement: Supplementary file 1 [file toxins-17-00284-s001.zip › Table S1.tif]
